# Management of hypopituitarism during pregnancy in patients with *PROP1*-related combined pituitary hormone deficiency: Review of the literature with a case report

**DOI:** 10.1007/s11102-025-01606-0

**Published:** 2025-12-08

**Authors:** Stella Pigni, Giulia Marsan, Marina Caputo, Chiara Mele, Madalina Elena Iftimie, Gabriela Rossi, Roberta Pasin, Paolo Marzullo, Gianluca Aimaretti, Flavia Prodam

**Affiliations:** 1 Department of Translational Medicine, University of Piemonte Orientale, Novara, Italy; 2 Department of Biomedical Sciences, Humanitas University, Pieve Emanuele (MI), Italy; 3 Department of Health Sciences, University of Piemonte Orientale, Novara, Italy; 4 Department of Endocrinology, “Carol Davila” University of Medicine and Pharmacy, Bucharest, Romania; 5 Gynecology and Obstetrics, Consultori Familiari ASST Fatebenefratelli-Sacco, Milan, Italy; 6 Gynecology and Obstetrics, Centro di Procreazione Medicalmente Assistista , Istituto Clinico Città Studi, Milan, Italy

**Keywords:** Pregnancy, Combined pituitary hormone deficiency, Hypopituitarism, Hormone replacement therapy

## Abstract

**Background:**

The management of pregnancy in patients with combined pituitary hormone deficiency (CPHD) represents a unique challenge due to the complex interplay of multiple pituitary hormone deficiencies, higher risk of feto-maternal complications, and lack of established evidence-based clinical guidelines. Though improvements in assisted reproductive techniques (ART) and multidisciplinary care over the last decades have increasingly enabled successful pregnancies in hypopituitary women, genetic causes of CPHD are rarely identified and data on pregnancy outcomes in affected women are scarce, underscoring the need for further case documentation to better inform clinical practice.

**Aims:**

We describe the case of a woman with genetically confirmed *PROP1*-related CPHD who achieved and completed a successful pregnancy through ART and tailored endocrine management throughout gestation and the postpartum period. This case report highlights the importance of preconception counseling, careful hormone replacement, and close monitoring and collaboration among expert endocrinologists, obstetricians, and neonatologists to optimize maternal and fetal outcomes in women with genetic CPHD. Furthermore, we provide a comprehensive literature review exploring key issues related to the main clinical and therapeutic challenges in the management of hypopituitarism during pregnancy, and particularly in the context of CPHD.

## Introduction

Combined pituitary hormone deficiency (CPHD) is a rare disorder characterized by the impaired production or secretion of at least two anterior pituitary hormones. This condition can be acquired or, more rarely, congenital resulting from genetic mutations in transcription factors critical for pituitary development, such as *PROP1*,* POU1F1*,* GLI2*,* HESX1*,* LHX3*,* LHX4*,* OTX2*,* SOX2* and* SOX3* [1–4]. Among these, the *PROP1* (Prophet of Pit-1) gene encodes for a pituitary-specific, paired-like homeodomain transcription factor involved in early pituitary development and required for determination, differentiation, and proliferation of anterior pituitary cells [3, 5, 6]. *PROP1* mutations represent the most frequent known genetic cause of CPHD, typically leading to sequential deficiencies of varying degree in growth hormone (GH), prolactin (PRL), thyroid-stimulating hormone (TSH), gonadotropins, and, less commonly, adrenocorticotropic hormone (ACTH), with age of onset ranging from infancy to adulthood [7–9].

Pregnancy in women with CPHD has been historically considered a challenging goal due to hypogonadotropic hypogonadism leading to anovulation and infertility and the complexity of managing multiple hormone replacements, in particular in genetic CPHD. Nonetheless, improvements in assisted reproductive techniques (ART) and multidisciplinary care over the last decades have increasingly enabled successful pregnancies in hypopituitary women, thus progressively raising questions about optimal endocrine management during pre-conception and gestation, along with concerns for the potential maternal and fetal complications [10].

One of the most debated issues is whether to discontinue or not GH replacement therapy (GHRT) during pregnancy. Although current guidelines recommend GHRT withdrawal once pregnancy is achieved [10, 11], some evidence suggests that GHRT may play a role in early gestation by supporting maternal metabolism, placental development, and fetal growth, especially prior to full establishment of placental GH (PGH) production [12–16].

The management of adrenal insufficiency (AI) during pregnancy adds further complexity, being hindered by the lack of dedicated guidelines, different cortisol and ACTH threshold values, higher risk of complications, and non-specific symptoms, which frequently overlap with those of pregnancy [17–19]. Physiologic adaptations of the hypothalamic-pituitary-adrenal (HPA) axis during gestation require careful titration of glucocorticoid dosage to avoid both under- and over-replacement, the former increasing the risk of adrenal crisis and the latter potentially contributing to adverse metabolic and obstetric outcomes [10, 18–20]. Yet, optimal dosing strategies remain undefined, particularly in patients with secondary AI, and current practice mainly relies on clinical judgment.

Similarly, adjusting thyroid hormone replacement during pregnancy in patients with central hypothyroidism is essential to ensure adequate fetal neurodevelopment. Although the stimulatory effect of placental beta-human chorionic gonadotropin (βHCG) on the TSH receptor may, at least in part, mitigate thyroid hormone needs in this context, such mechanism might be insufficient in patients with chronic TSH deficiency, potentially resulting in the need for significant levothyroxine dose escalation to maintain maternal euthyroidism [10, 21].

Further complicating the clinical picture, pregnancy in hypopituitary women has been associated with increased risks of miscarriage, cesarean section (C-section), small-for-gestational-age neonates, and postpartum complications [22, 23], though favorable outcomes are achievable with meticulous planning, multidisciplinary coordination, and individualized care [10].

To date, few reports have described successful pregnancies in women with congenital genetic CPHD [24–27], underscoring the need for further case documentation to better inform clinical practice [28].

Herein, we describe the case of a woman with genetically confirmed *PROP1*-related CPHD who achieved a successful pregnancy through ART and tailored endocrine management throughout gestation and the postpartum period. Furthermore, we provide a comprehensive literature review exploring key issues related to the main clinical and therapeutic challenges in the management of hypopituitarism during pregnancy in this rare but increasingly relevant setting.

## Case presentation

### Baseline characteristics

A Caucasian woman with a diagnosis of hypopituitarism (GH deficiency, central hypothyroidism, secondary AI, and hypogonadotropic hypogonadism) resulting from a homozygous pathogenic variant c.150del (p.Arg53AspfsTer112) in the *PROP1* gene was first referred to our Endocrinology clinic at the age of 19 years. She was diagnosed with genetic CPHD during evaluation for growth retardation at the age of 6 years, and received full hormone replacement for all deficiencies, reaching normal stature and complete pubertal development. Pubertal induction was initiated at the age of 13 years with transdermal estradiol patches, followed by the introduction of sequential progesterone once breakthrough bleeding occurred, after approximately two years. From the age of 16 years, the patient reported regular menstrual cycles under combined transdermal estrogen and cyclic progesterone therapy.

### Management during preconception

At the age of 26 years, in her pursuit of pregnancy, the patient was referred to a Fertility clinic where part of the preconception counseling and initial gynecologic assessment were performed. She had been attempting conception for approximately 3 years. Multidisciplinary preconception counselling included genetic evaluation to discuss hereditary risks and possible implications for conception and offspring, review and optimization of hormone replacement therapy, and assessment of potential maternal and fetal risks. Hormone evaluations revealed undetectable gonadotropins levels (FSH and LH < 0.3 IU/L) and normal anti-Müllerian hormone (AMH) levels. A vaginal ultrasound showed normal ovarian and uterine size. Sono-hysterography initially suggested a possible tubal factor infertility, though this was not confirmed by hysterosalpingography, which demonstrated bilateral tubal patency. Given her young age and confirmed tubal patency, first-line treatment was initiated with controlled ovarian stimulation. The regimen involved incremental menotropin dosing, starting at 37.5 IU and increasing to 75 IU, followed by hCG administration. This stimulation protocol, combined with timed intercourse, was repeated three times without achieving conception, with the first cycle cancelled due to inadequate ovarian response to stimulation. Due to the limited ovarian response and failure to conceive with first-line approach, the patient was referred for second-line treatment with in vitro fertilization (IVF). An initial stimulation using follitropin delta did not produce adequate follicular development. A subsequent protocol employing recombinant follitropin alfa combined with lutropin alfa resulted in nine developing follicles. Final oocyte maturation was triggered with recombinant hCG alfa, followed by transvaginal oocyte retrieval (pick-up). Five oocytes were retrieved, resulting in fertilization of one embryo, three blastocysts, and one unfertilized oocyte. The first two blastocyst transfers both resulted in first-trimester miscarriage. A third transfer performed three months later led to a successful pregnancy.

Prior to pregnancy, the patient was on treatment with modified-release hydrocortisone (20 mg/day), levothyroxine (100 µg/day), and somatotropin (3.5 mg/week). Prior to ovulation induction she was also receiving transdermal estradiol (50 µg patch applied twice a week) plus sequential micronized progesterone (100 mg/day for 14 days of each 28-day cycle), which ensured regular menstrual cycles. The levothyroxine dose was then adjusted in preparation for IVF, increasing the dosage to 100–125 µg on alternate days. Upon pregnancy confirmation, GHRT was discontinued and modified-release hydrocortisone was replaced with the standard formulation three times a day. The initial recommended total daily dose of hydrocortisone was 17.5 mg/day, corresponding to the patient’s previous regimen before switching to the modified-release formulation; however, this was soon self-adjusted by the patient to 20 mg/day and subsequently maintained given the adequate clinical and biochemical control. At the time of conception, hypopituitarism was well compensated (sodium 140 mEq/L, potassium 4.1 mEq/l; insulin-like growth factor 1 (IGF-1) 160.3 ng/mL, normal range 107.8–246.7 ng/mL; fT4 11.7 pg/mL, normal range 9.2–16.8 pg/mL; fT3 3.8 pg/mL, normal range: 2-4.4 pg/mL). She was normal weight (weight 50 Kg, height 158 cm, body mass index 20 Kg/m^2^) and had normal blood pressure and glucose levels.

### Management during pregnancy

Throughout gestation, biochemical monitoring included thyroid function (fT3, fT4) and electrolytes levels (sodium, potassium), while clinical monitoring focused on symptoms (such as fatigue), glucose levels (assess through venous sampling, glucometer readings, and continuous glucose monitoring (CGM)), blood pressure, and weight measurements every two-to-three week. Adherence to hormone replacement therapy was also systematically reviewed at each follow-up visit and the patient consistently reported good compliance with all prescribed regimens.

During the first trimester, sex hormones were managed with administration of oral estradiol valerate and progesterone given both intravaginally and subcutaneously (on alternate days), in accordance with gynecologic recommendations. During the late second and third trimesters, hydrocortisone dosage was gradually increased (from 20 mg/day up to 30 mg/day at the end of the third trimester) to maintain clinical stability, normal blood pressure and electrolytes levels. Indeed, this progressive dose adjustment was prompted partly by the patient’s complaints of fatigue, but also by biochemical findings showing a trend toward lower sodium and higher potassium levels (although remaining within normal limits) as well as a tendency to hypoglycemia for which CGM had been implemented. Levothyroxine dosage was also incremented up to a maximum dose of 150 µg/day in the second trimester and 200 µg/day in the third trimester, targeting fT4 levels in the upper half of the normal range. At 26 weeks of gestation, a 75-g oral glucose tolerance test (OGTT) ruled out gestational diabetes. As a precaution, the patient also underwent a cardiology evaluation, including electrocardiogram and echocardiography, with normal results.

**Fig. 1:**
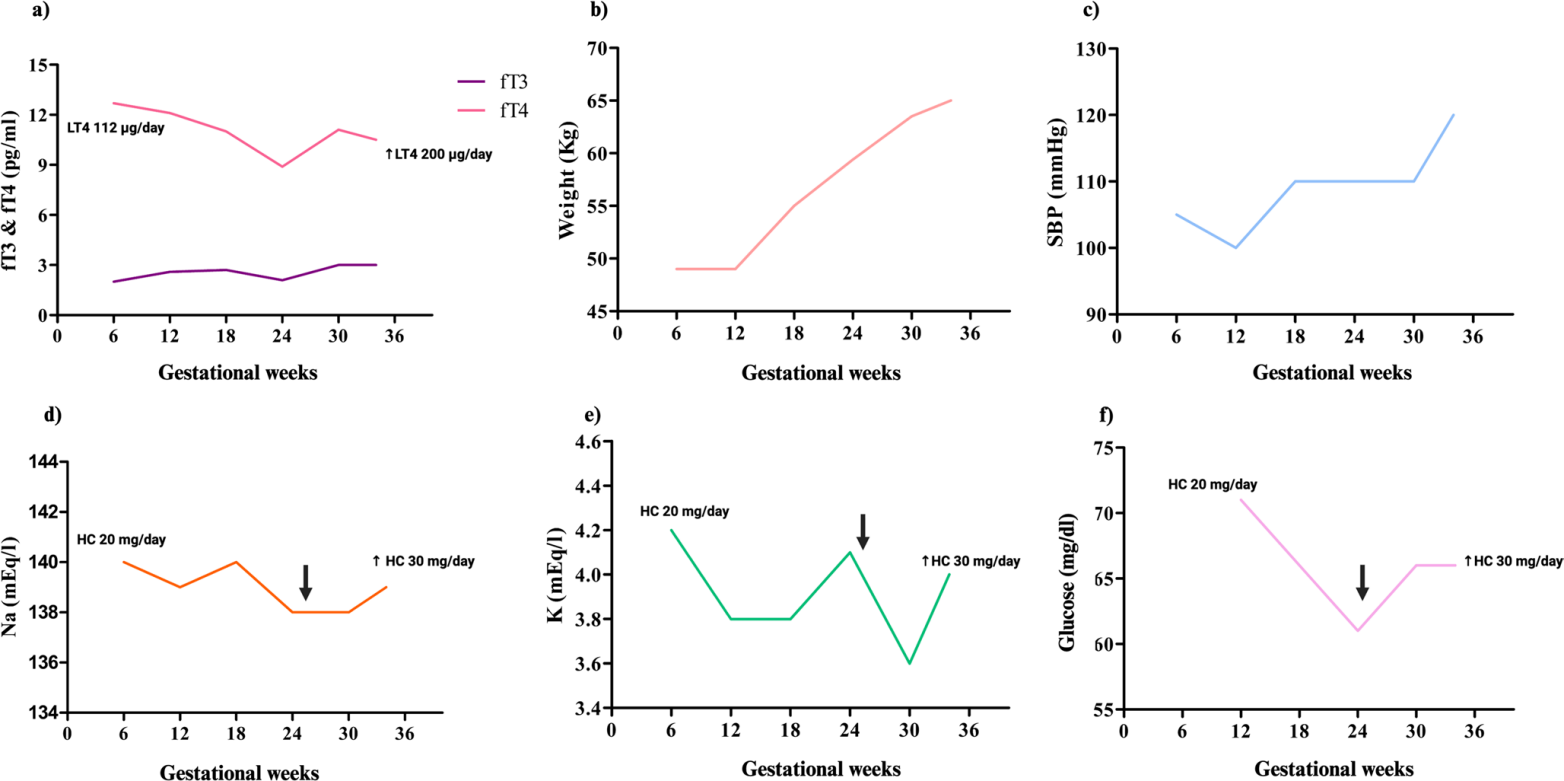
Trend of main clinical and biochemical parameters monitored during pregnancy. The panels represent: a) free thyroid hormones (fT3, fT4); b) maternal weight; c) systolic blood pressure; d) sodium levels; e) potassium levels; f) blood glucose. Abbreviations: LT4, levothyroxine; HC, hydrocortisone; Na, sodium; K, potassium; SBP, systolic blood pressure. Arrows mark the time when hydrocortisone dose escalation began. fT3 normal range: 2-4.4 pg/mL, fT4 normal range 9.2-16.8 pg/mL

Over the entire course of pregnancy, the patient remained clinically stable and fetal growth was appropriate for gestational age. Final maternal weight before delivery was 63 Kg. The mammary gland also progressively increased. Trend of main clinical and biochemical parameters monitored throughout pregnancy is presented in Fig. 1.

Delivery was planned at a tertiary-level obstetrics and neonatal intensive care unit to ensure multidisciplinary management of hypopituitarism for both the mother and, eventually, the newborn. At 39 weeks, precautionary C-section was performed, resulting in the delivery of a healthy male infant (normal Apgar score, birth weight 3600 g). The delivery was carefully coordinated with the obstetric/gynecologist team, which had been provided with a steroid infusion protocol during and after surgery (two intravenous (iv) boluses of 50 mg and 100 mg hydrocortisone, followed by a continuous hydrocortisone infusion of 200 mg over 24 h). In addition, on postpartum days two and three, administration of two additional iv hydrocortisone boluses was indicated due to moderate hyponatremia (sodium 128 mEq/L), low blood pressure, and fatigue, with good clinical and biochemical response. The patient was subsequently transitioned to oral hydrocortisone before discharge.

### Management during postpartum

Both mother and newborn were discharged from the hospital 4 days after delivery. The infant was in good condition, with initial clinical evaluations showing normal growth and genital development, as well as normal blood glucose and electrolytes levels, and thyroid function, while genetic testing confirmed the heterozygosity for the known maternal pathogenic *PROP1* variant. As expected, despite appropriate mammary gland enlargement during gestation, the patient developed agalactia and PRL levels confirmed the suspected deficiency (23 mU/L, range 102–496 mU/L), which precluded breastfeeding.

**Fig. 2:**
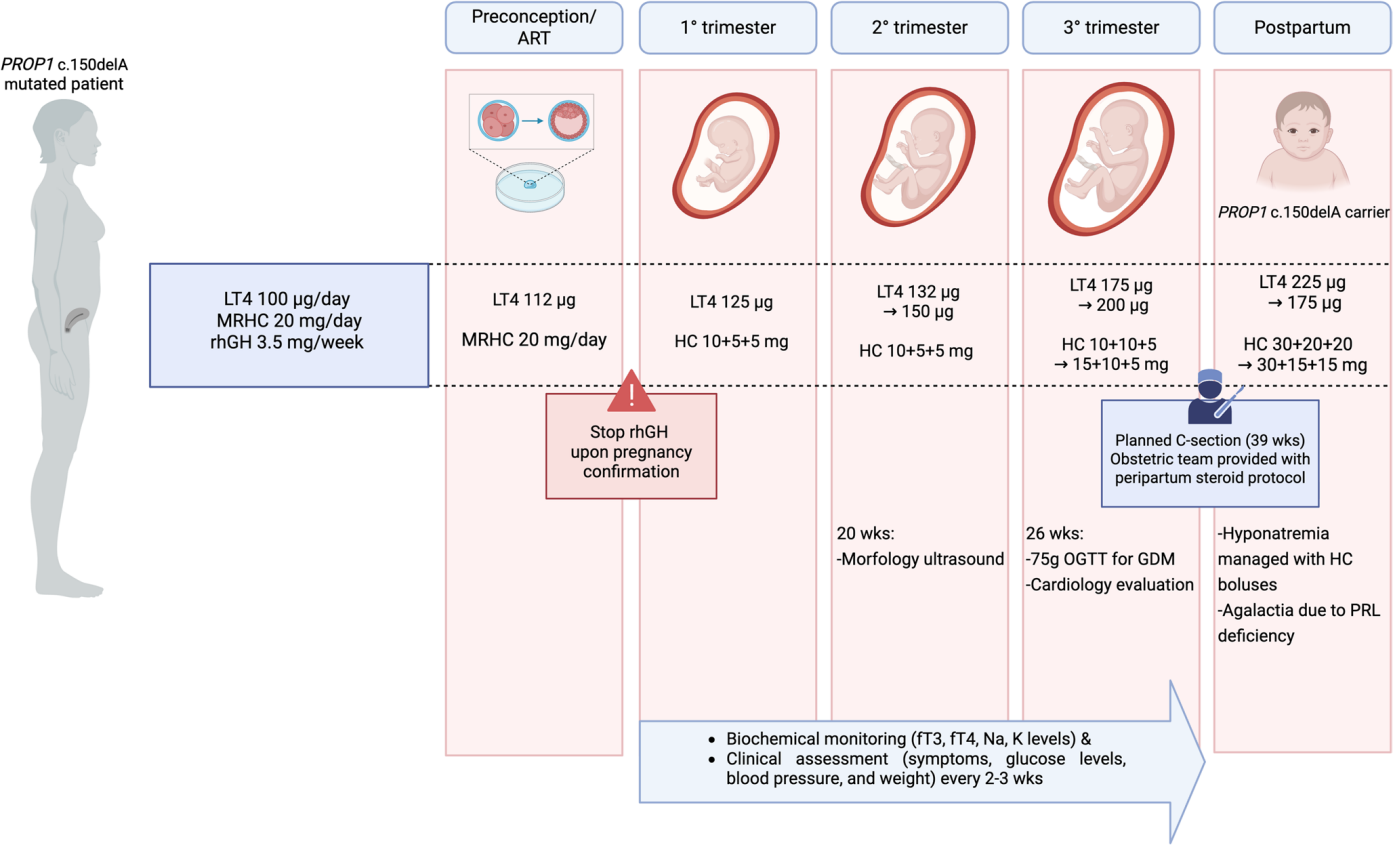
Overview of clinical and biochemical assessment, and therapeutic adjustments made in preparation for assisted reproduction, during the trimesters of pregnancy and in the postpartum period. *Created with Biorender.com. *Abbreviations: LT4, levothyroxine; HC, hydrocortisone; MRHC, modified-release hydrocortisone; rhGH, recombinant human growth hormone; ART, assisted reproductive techniques; wks, weeks; Na, sodium; K, potassium; GDM, gestational diabetes mellitum; C-section, Caesarian section; PRL, prolactin

In the puerperium, further adjustments of both levothyroxine and hydrocortisone doses were required, with hydrocortisone temporarily doubled due to persistent fatigue and general malaise, which led to an emergency department visit and urgent endocrinological evaluation, and a further increase in levothyroxine (up to 225 µg/day). Therapeutic adjustments during pregnancy are comprehensively summarized in Fig. 2.

At 6 months postpartum, the patient remained on a doubled dose of hydrocortisone therapy due to tapering difficulties, while thyroid hormone dosage was gradually decreased to 175 µg/die, still higher than before pregnancy, but likely related to the maintainance of an increased body weight. Of note, the tapering to hydrocortisone standard dose required more than 1 year. Nevertheless, despite the heightened glucocorticoid dose, the patient showed no clinical or biochemical signs of glucocorticoid overreplacement, suggesting an increased cortisol requirement and adequacy of the replacement regimen during this period. Oral estrogen-progestin replacement therapy was reinitiated one month after delivery, while GHRT was resumed 2 months later. Furthermore, the patient was referred for nutritional counseling due to difficulties losing the weight accrued during pregnancy, and a structured dietary plan was implemented to support weight management.

## Discussion

The management of pregnancy in patients with CPHD represents a unique challenge due to the complex interplay of multiple pituitary hormone deficiencies, higher risk of feto-maternal complications, and lack of established clinical guidelines. Moreover, genetic causes of CPHD are rarely identified and data on pregnancy outcomes in affected women are scarce, further complicating the clinical decision-making in this context.

Herein we described a case of successful pregnancy in a patient with a c.150delA mutation in the *PROP1* gene, which is one of the most common variants associated with both familiar and sporadic congenital CPHD [2, 3, 29]. In these patients, GH deficiency (GHD) usually develops early in life, followed by additional deficiencies in TSH, PRL, gonadotropins, and, less frequently, ACTH [2, 26]. However, age and order of onset of the various pituitary hormone deficiencies may vary, even among individuals with the same pathogenic variant. For instance, spontaneous puberty with subsequent progressive decline of gonadotroph function has been reported in some patients carrying the *PROP1* variant c.150delA [7]. Nonetheless, the loss of gonadotropin secretion almost invariably becomes evident by late adolescence/young adulthood [2, 7, 26].

Women with impairment of pituitary gonadotroph function often required ART for conception. Ovarian stimulation with exogenous gonadotropins is the recommended strategy for ovulation induction, followed by either timed intercourse (TI) or ART depending on the presence of other infertility factors. IVF may also be an alternative when conception fails after repeated cycles of successful ovulation induction and TI [10, 30, 31]. Although advances in these techniques in recent years have led to increased pregnancy rates in hypopituitary women, data in patients with CPHD are still scarce as most of the published studies involved women with isolated hypogonadotropic hypogonadism. Of note, conception in these patients may be hindered not only by the absence of gonadotropins, but also by disturbances in the secretion of other pituitary hormones [10].

Early retrospective studies in patients with pan- or partial hypopituitarism of varying etiologies receiving gonadotropins for ovulation induction reported a relatively high number of cancelled cycles, suggesting a low ovarian response to stimulation. The pregnancy rate ranged between 47 and 76%, compared with over 81% in women with isolated hypogonadotropic hypogonadism, with a live birth rate of about 42–79% [10, 32, 33]. A trend towards poorer pregnancy outcomes in patients with a childhood onset of hypopituitarism was also observed [33]. Conversely, in a more recent prospective study on 5 women with childhood onset CPHD (including one woman with *GLI2* gene mutation), Correa et al. reported a 100% pregnancy rate after ovarian stimulation and intrauterine insemination/IVF [26]. Patients’ young age at fertility treatment, as well as the advances in ART over time, and adequate hormone replacement for all deficiencies, including GHD, might have influenced these positive outcomes [26]. It should also be noted that all patients ovulated and achieved pregnancy within one to five cycles of ovulation induction over a treatment period of approximately two years [26], suggesting that the reproductive outcome observed in our patient was within expectations for women with optimally treated CPHD. Interestingly, reduced antral follicle count (AFC) and low AMH concentrations have been frequently observed in women with CPHD, particularly those with severely suppressed gonadotropin levels [26, 34, 35], and have been associated with longer ovarian stimulation durations in this series [26]. Nevertheless, though our patient had undetectable gonadotropins levels since childhood, her AMH levels were normal. These findings underscore the need for further research to identify reliable predictors of ovarian response in patients with congenital CPHD and provide tailored ovarian stimulation protocols for this complex population.

To our knowledge, there is only another case report describing successful pregnancies in two young women carrying a homozygous GA296del mutation of the *PROP1* gene. Both patients had GH, TSH, gonadotropins, and PRL deficiency and achieved ovulation and pregnancy with the use of only gonadotropins and levothyroxine treatment [25]. Notably, GHRT was not required to conceive and both patients delivered normal, full-term newborns [25]. Even if normal pregnancies have been reported in some patients with untreated GHD and in women with Laron-type dwarfism, suggesting that the somatotrophic axis may not be essential for ovulation and conception [14, 24, 25, 36, 37], compelling evidence has demonstrated that adding GHRT to ovulation induction and ART protocols can improve pregnancy rates and outcomes in hypopituitary women seeking fertility [26, 37–39]. In this view, once our patient had expressed the desire to conceive, all replacement therapies were maintained and further adjusted as appropriate in preparation for ART, including GHRT.

Another ongoing controversy in the management of hypopituitarism during pregnancy involves whether and when GHRT should be discontinued. Indeed, while the need for adequate GH replacement prior to conception has been widely recognized in clinical practice, GHRT is not approved for use during pregnancy and current guidelines recommend its discontinuation once pregnancy is confirmed [10, 11]. This recommendation relies on the increasing production of PGH throughout pregnancy, which replaces pituitary GH in stimulating maternal IGF-1 production, and the lack of data from randomized controlled trials (RTCs) on the effects and safety of GHRT during conception and pregnancy [10, 11]. Moreover, in a study on a large cohort of women with GHD (either isolated or combined with other pituitary deficits), Vila et al. found no relationship between GHRT regimens and pregnancy outcomes [39]. Although no major abnormalities have been observed in pregnancies of untreated GHD patients, some evidence exists suggesting potential benefits of continuing GHRT during pregnancy, particularly in optimizing maternal metabolism and psychological well-being, as well as placental development and fetal growth [14–16]. Interestingly, Aulinas et al. recently reported a high rate of excessive gestational weight gain and C-section in a cohort of hypopituitary women who discontinued GHRT during pregnancy [21]. Furthermore, PGH production starts from the fifth week of gestation and reaches its peak in the second half of pregnancy, thus leaving a period in which maternal GHD may impact maternal and fetal outcomes [10, 12, 40]. Hence, some authors advocate to continue GHRT at the pregestational dose in the first gestational trimester, then halve the dose during the second trimester, and interrupt the treatment at the beginning of the last trimester [16, 41]. Another proposed strategy is to maintain the GRHT throughout pregnancy while monitoring IGF-1 levels [26]. Even if these protocols have been proven safe in some case series, case reports, and retrospective analyses [16, 26, 41–43], they have not been validated in RTCs and additional data on the safety of GHRT during pregnancy are needed. Therefore, in accordance with current recommendations from the Endocrine Society [11], we discontinued GHRT in our patient as soon as pregnancy was confirmed. Despite discontinuation, no negative influences on maternal weight, metabolic profile or psychological well-being were observed, and fetal growth and birth weight were normal, suggesting that individualized approaches may be necessary based on the clinical context and patients’ wish.

One of the most challenging aspects of hypopituitarism during pregnancy is the management of glucocorticoid replacement in patients with central AI. During gestation, the HPA axis undergoes significant changes, resulting in a physiologic hypercortisolemic state that helps modulate the immune system and support fetal development [10, 19]. Therefore, glucocorticoid replacement in this period requires careful adjustments to mimic the physiology of cortisol production and accommodate the increased maternal hormone needs.

Apart from knowledge on the physiological HPA axis changes during gestation in healthy women, current evidence on the management of AI during pregnancy mainly derives from studies in patients with primary AI, and there are no dedicated guidelines for the optimal glucocorticoid regimen to use, thus making personalized adjustments necessary [10, 18–20]. Hydrocortisone is the glucocorticoid of choice during pregnancy, as it becomes inactivated by placental 11β-hydroxysteroid dehydrogenase type 2, thus minimizing the risk of fetal exposure. Typically, the dose of hydrocortisone should be increased by 20–40% during the third gestational trimester [10, 18–20, 28]. The glucocorticoid regimen should be adjusted based on clinical and biochemical assessments, carefully monitoring the patient for signs and symptoms of under- (i.e. hyponatremia, hyperkaliemia, hypoglycemia, adrenal crisis) or over-replacement (i.e. excessive weight gain, hypertension, gestational diabetes) at least once every trimester [10, 18–20, 28]. Importantly, glucocorticoid excess has been also associated with adverse fetal outcomes, including pre-term delivery, low birth weight and increased cardiometabolic risk later in life [19, 44–46]. For adrenal crisis prevention, intravenous stress doses of hydrocortisone should be given during labor/delivery and C-Sect. (50 mg at second stage of labor and 100 mg IV preoperatively followed by continuous infusion or boluses every 6–8 h postoperatively for C-section) [10, 18–20, 28]. In our case, upon pregnancy confirmation the patient was transitioned from modified-release hydrocortisone to the standard formulation, and the dosage was split into 3 times per day to allow for a more physiologic replacement and more precise dose titration [10, 47]. Her glucocorticoid regimen was then adjusted incrementally starting from the last gestational trimester up to 50% of pre-pregnancy dose, and iv hydrocortisone was administered during C-section as per current guidelines recommendations [10, 11]. Following this approach, she remained clinically stable over the course of pregnancy, which was uneventful. However, despite these proactive measures she experienced post-partum hyponatremia and long-lasting general malaise. While in absence of intercurrent illnesses it is generally recommended to double the usual glucocorticoid dosage for a few days after delivery before tapering back to the normal pre-pregnancy dose (within 1 week) [10, 11, 19, 20], our patient required significant implementation of the hydrocortisone dose for several weeks after childbirth before clinical stability was re-established. Furthermore, the tapering process required more than one year, which could reflect the possible occurrence of a “glucocorticoid withdrawal syndrome” after prolonged exposure to supraphysiologic doses of glucocorticoids. In addition, several potential hormonal replacement interactions may have contributed to this pattern, such as increased cortisol metabolism (at least in part through stimulation of 11β-hydroxysteroid dehydrogenase type 2) due to enhanced levothyroxine therapy [11, 18], and the effects of GH substitution, as GH can reduce the activity of 11β-hydroxysteroid dehydrogenase type 1, thus decreasing cortisol levels [48, 49]. Therefore, our experience emphasizes the importance of close patient monitoring to ensure proper, individualized glucocorticoid dosage adjustment throughout gestation, including the post-partum period. Moreover, a collaborative approach between the endocrinologist and the obstetric team, including providing a therapeutic plan regarding glucocorticoid coverage during the labor/delivery or C-section is of utmost importance to minimize the potential risk of adrenal crisis [50]. Notably, recent evidence has suggested that a systematic dose increase in glucocorticoid replacement may not be necessary in all patients with secondary AI during pregnancy, and that some women may require smaller dose increases than previously recommended [21, 50]. Aulinas et al. also hypothesized that the stop of GHRT could have contribute to the lower requirements of hydrocortisone observed in their case series (including 5 women with ACTH-deficiency) [21]. However, discontinuation of GHRT seem to have had little to no impact in our case. Although in some hypopituitary women the adrenals may at least in part respond to placental corticotropin-releasing hormone (pCRH) and ACTH stimulation [19, 51], this may not apply to patients with long-standing ACTH deficiency, such as those with congenital CPHD and early onset AI. Furthermore, a recent multicenter survey found no significant differences in hydrocortisone equivalent dosage during the second and third trimester of pregnancy between women with Addison’s disease and secondary AI [50].

Regarding thyroid replacement, ensuring euthyroidism is of utmost importance for the normal fetal cognitive development and levothyroxine requirements typically increase during pregnancy. Clinical guidelines from the American Thyroid Association (ATA) for the management of thyroid disease during pregnancy recommend increasing the levothyroxine dose of 20–50% at the beginning of pregnancy [52]. Conversely, according to current guidelines from the Endocrine Society and the European Thyroid Association (ETA), there is no standard regimen for dose escalation in pregnant patients with central hypothyroidism, but strict follow up measuring fT4 levels every 4–6 weeks is recommended [11, 53]. Recently, a position statement on the diagnosis and management of congenital CPHD in adults has proposed a 30% increase in thyroid hormone dosage as soon as pregnancy is confirmed [28]. Throughout gestation, fT4 concentrations should be measured every 4–6 weeks, targeting these levels in the upper half of the normal range. After delivery, levothyroxine dosage can be reduced to pre-pregnancy levels [28]. Under physiologic condition, placental βHCG can directly stimulate the TSH receptor due to its structural similarity with TSH, thereby increasing thyroid hormone production, while reducing TSH levels, particularly during the first trimester [10, 54]. Therefore, it has been speculated that women with TSH deficiency and a functioning thyroid gland may have lower levothyroxine requirements during pregnancy compared with patients with primary hypothyroidism [10]. By contrast, some recent case series have reported a high average increase (71.7–100%) in levothyroxine doses during pregnancy in women with panhypopituitarism of varying etiologies [21, 27]. In line with this evidence, in our case levothyroxine dosage was progressively adjusted based on fT4 levels, with a total increase of about 78% of pre-pregnancy dose. Altogether, these findings suggest that the response to the TSH-like activity of βHCG may vary in hypopituitary women during pregnancy, potentially being lower in patient with chronic thyrotropic deficiency. As with glucocorticoid replacement, this could result in a higher demand for thyroid hormones throughout gestation in these patients than usually assumed. Of note, our patient required an increased dose of levothyroxine even in the postpartum period. This could be partly explained by postpartum weight retention; however, the resumption of GHRT after delivery may also have contributed, as GH can decrease circulating fT4 levels [49, 55]. In addition, several other factors may have played a role in increasing levothyroxine requirements during pregnancy and postpartum, including altered intestinal absorption (potentially affected by concomitant use of supplements, such as iron and calcium preparations commonly administered during pregnancy), expanded maternal plasma volume diluiting thyroid hormone concentrations, estrogen-induced rises in thyroxine-binding globulin (TBG) reducing free hormones levels (possibly further enhanced by the reintroduction of oral estrogen therapy postpartum), increased inactivation of thyroxine and triiodothyronine by type 3 iodothyronine deiodinase (which is widely expressed in the human fetoplacental unit and uterus), and changes in hepatic and renal clearance of thyroxine [10, 11, 52, 53, 56].

Pregnancy in hypopituitary patients is reported to carry a high risk of maternal-fetal complications, including higher rates of miscarriage, fetal malpresentation, small for gestational age (SGA) rate, and postpartum hemorrhage [10, 22, 23]. In the case report by Voutetakis et al., both pregnancies in patients with *PROP1*-related CPHD went uneventfully to term. One patient delivered by C-section and one patient vaginally, and both newborns were healthy, with normal Apgar scores, and normal birth weights [25]. Considering other recent case series in women with CPHD, Correa et al. reported only one miscarriage, one case of intrauterine growth restriction requiring neonatal intensive care, and one fetal malpresentation (breech lie) [26]. Conversely, Aulinas et al. did not observed any miscarriage, nor obstetrical or fetal complications, apart from excessive maternal weight gain [21]. Interestingly, in both studies the authors reported a high rate of C-section (60%) [21, 26]. In our case, the patient underwent three blastocyst transfers, resulting in 2 spontaneous first trimester miscarriages before a successful pregnancy was achieved. Although there were no major obstetrical or fetal complications over the course of pregnancy, delivery was planned at 39 weeks via C-section due to concerns regarding adrenal stress and potential labor complications. Indeed, even if data on operative deliveries are often incomplete, the high C-section rate reported in hypopituitary women may, at least in part, reflect obstetricians’ recommendations rather than strict medical necessity [21, 23, 26].

It is also noteworthy that despite appropriate mammary gland enlargement during gestation a PRL deficiency was confirmed postpartum, consistent with patient’s CPHD [57]. Both the patient and the obstetric team had been counseled in advance regarding the likelihood of agalactia, allowing prompt implementation of alternative feeding methods.

In conclusion, pregnancy in patients with congenital CPHD is a clinical challenge, but it can be safely managed with appropriate multidisciplinary care in specialized centers. Preconception counseling, careful hormone replacement, and close monitoring during pregnancy and the postpartum period are crucial to optimize maternal and fetal outcomes. In particular, the management of AI remains challenging due to the lack of dedicated guidelines, with dose adjustments relying primarily on clinical monitoring and best practice recommendations. Potential interactions between multiple hormone deficiencies and their respective replacement therapies may further complicate management, as these could dynamically influence treatment requirements throughout gestation and beyond. Integrated collaboration among expert endocrinologists, obstetricians, neonatologists, and anesthesiologists, supported by a clear management plan for each phase (from preconception optimization, through pregnancy and delivery, to the postpartum period) are key determinants for ensuring timely, coordinated interventions and minimizing risks for both the mother and the newborn in these complex cases.

## Data Availability

No datasets were generated or analyzed during the current study.
